# Cardio-Renal Effects of Short-Term Fructose Treatment in Hypertensive Rats: Focused on NO/ROS Balance

**DOI:** 10.33549/physiolres.935724

**Published:** 2025-12-01

**Authors:** Olga PECHANOVA, Radoslava BULKOVA, Stanislava VRANKOVA, Jana KLIMENTOVA, Zuzana GALANDAKOVA, Martina CEBOVA

**Affiliations:** 1 Institute of Normal and Pathological Physiology, Centre of Experimental Medicine, Slovak Academy of Sciences, Bratislava, Slovak Republic; 2 Institute of Pathophysiology, Faculty of Medicine, Comenius University, Bratislava, Slovak Republic

**Keywords:** Fructose, Kidney, Heart, Oxidative Stress, Lipids, Nitric Oxide

## Abstract

Short-term fructose exposure may perturb the nitric oxide (NO)/reactive oxygen species (ROS) balance before hemodynamic changes development. Male Wistar Kyoto (WKY) and spontaneously hypertensive rats (SHR) rats received 10 % fructose in drinking water for 3 weeks or remained on tap water. We assessed systolic blood pressure (tail-cuff), plasma lipid levels, tissue conjugated diene concentrations, protein expression of NADPH oxidase, NF-κB, and SOD (Western blot), and total NO synthase (NOS) activity ([^3^H]-L-arginine to [^3^H]-L-citrulline). Fructose did not change blood pressure in either strain, but increased kidney-to-body-weight ratio in SHR. In WKY, plasma HDL level decreased; in SHR, total cholesterol, VLDL, and triglycerides increased. Conjugated diene concentration increased in the kidney of WKY but not in the heart. Fructose upregulated renal NADPH oxidase and downregulated renal SOD in SHR, with no change in cardiac NADPH oxidase. NF-κB protein expression did not change in either tissue. NOS activity decreased in the heart and kidney of WKY and in the kidney of SHR. We can conclude that even moderate, short-term fructose intake induces strain-dependent dyslipidemia and an early shift of the renal redox milieu toward oxidative stress, accompanied by reduced NOS activity, while leaving blood pressure unchanged. The kidney appears more susceptible than the heart, particularly in the hypertensive background, highlighting the NO/ROS axis as an early target for intervention.

## Introduction

Fructose is a monosaccharide that is widely available in natural food sources such as fruits, vegetables, and honey. However, in modern diets most exposure comes from added sugars (sucrose or high-fructose corn syrup) in processed foods and sugar-sweetened beverages. Large prospective analyses and recent global burden assessments consistently link higher intake of sugar-sweetened beverages with increased risk of type 2 diabetes and cardiovascular disease [1,2].

Fructose metabolism differs fundamentally from that of glucose. After intestinal absorption, primarily via the fructose-specific transporter GLUT5 with context-dependent apical recruitment of GLUT2, fructose enters the portal circulation and is rapidly phosphorylated by ketohexokinase (KHK) [3,4]. Unlike glucose, acute fructose ingestion elicits minimal insulin and leptin responses, a hormonal profile that may favor positive energy balance [4]. The small intestine is now recognized as a major site of first-pass fructose metabolism that can shield the liver from fructose exposure at modest doses. When this capacity is exceeded, fructose “spills over” to the liver and colonic microbiota, amplifying metabolic perturbations [5]. In hepatocytes, unregulated fructolysis bypasses phosphofructokinase control, driving de novo lipogenesis, hypertriglyceridemia, and ectopic fat accumulation through ChREBP/SREBP-1c–dependent programs [6].

A key biochemical consequence of rapid ketohexokinase-mediated fructolysis is ATP depletion with uric acid generation. Elevated hyperuricemia is mechanistically linked to endothelial dysfunction through nitric oxide (NO) depletion and redox-inflammatory signaling, thereby connecting high fructose exposure to cardiometabolic risk [7,8]. Gersch *et al.* [9] demonstrated that uric acid reacts directly with NO in a rapid, irreversible reaction, resulting in the formation of 6-aminouracil and depletion of NO.

Further evidence demonstrates that high-fructose diets cause disruption in the ratios of acetyl-coenzyme A to coenzyme A and nicotinamide adenine dinucleotide [10,11]. Fructose may also elevate concentrations of angiotensin II [12] - a potent activator of NAD(P)H oxidase contributing to the production of reactive oxygen species (ROS) [13,14]. An increase in cellular production of ROS leads to activation of intracellular signaling pathways [15], including the transcription factor nuclear factor-κB (NF-κB), which regulates the expression of numerous genes, including those encoding superoxide dismutase (SOD), endothelial NO synthase (eNOS), and inducible NO synthase (iNOS) [16,17].

The kidney is an important site of fructose disposal, and recent studies suggest that metabolic syndrome is a sequential risk factor for chronic kidney disease [18,19]. The kidney metabolizes fructose (proximal tubule KHK/GLUT5 expression) and is vulnerable to fructose-induced oxidative stress, inflammation, and fibrosis - pathways relevant to chronic kidney disease [18].

Given the interplay between oxidative stress and NO bioavailability in cardiovascular-renal pathology, we investigated whether short-term fructose intake alters oxidative status and NO production in the heart and kidney of normotensive and spontaneously hypertensive rats.

## Material and Methods

### Chemicals and drugs

All the chemicals used were purchased from Sigma Chemicals Co. (Germany) when not specified.

### Animals

In this study, 24 male 6-week-old rats were used. Six Wistar Kyoto rats (WKY) and six spontaneously hypertensive rats (SHR) were taken as controls (drinking tap water), whereas the remaining rats were given free access for 3 weeks to 10 % fructose solution in drinking water. All animals were kept under standard laboratory conditions (12 h light, 12 h darkness, 22±2 °C, pelleted ST-1 diet, drinking *ad libitum*). All procedures and experimental protocols were approved by the Ethical Committee of the Institute of Normal and Pathological Physiology SAS and the State Veterinary and Food Administration of the Slovak Republic (3260/12-221).

### Blood pressure and weight parameters

Systolic blood pressure (SBP) was recorded weekly by non-invasive tail-cuff plethysmography in conscious, warmed and gently restrained rats. For each session, three consecutive readings were obtained and averaged. Upon completion of the treatment period, the animals were euthanized, and their body weight (BW), heart weight (HW), and kidney weight (KW) were recorded. The relative heart weight and relative kidney weights were determined by calculating the HW/BW and KW/BW ratio, respectively.

### Lipid profile

Blood plasma was collected to measure the level of total cholesterol (CHOL), triglyceride (TG), low density lipoprotein (LDL), very low density lipoprotein (VLDL) and high density lipoprotein (HDL) (ab65390, Abcam, Cambridge, UK).

### Conjugated diene concentration

To determine conjugated diene (CD) concentrations, heart and kidney tissues were homogenized in 15 mmol/dm^3^ EDTA and 4 % NaCl, as described previously [20]. Briefly, lipid extraction was performed using a mixture of chloroform and methanol (1:1). The chloroform layer was evaporated under a nitrogen atmosphere, and the residue was dissolved in cyclohexane. CD concentrations were measured spectrophotometrically at a wavelength of 233 nm using the NanoDrop 2000c UV-Vis spectrophotometer (Thermo Fisher Scientific, Waltham, MA, USA).

### Western Blot Analysis

Heart and kidney tissue samples were homogenized, and Western blot analysis was conducted following a previously described protocol [21]. Briefly, membranes were incubated overnight at 4 °C with the following primary antibodies: anti-NADPH oxidase 4 (1:2000, Abcam, ab154244), anti-NF-κB p65 (1:1000, Cell Signaling, 6956), anti-SOD1 (1:3000, Abcam 13498), and anti-GAPDH (1:5000, Abcam, ab201822) as a loading control. Subsequently, the membranes were incubated for 2 h at room temperature with a peroxidase-conjugated secondary goat anti-rabbit antibody (1:5000, Abcam, ab97051).

Protein bands were visualized using an enhanced chemiluminescence system (ECL, Bio-Rad, CA, USA) and quantified with a ChemiDoc™ Touch Imaging System (Image Lab™ Touch software, Bio-Rad, Hercules, CA, USA). Band intensities were normalized to GAPDH as a loading control for heart tissue samples.

### Total NO Synthase (NOS) activity

Total nitric oxide synthase (NOS) activity was quantified in crude heart and kidney homogenates by measuring the conversion of [^3^H]-L-arginine to [^3^H]-L-citrulline (ARC, Saint Louis, MO, USA), as described previously [22]. Briefly, 50 μL of 20 % tissue homogenate was incubated in a reaction mixture containing 0.5 M Tris-HCl (pH 7.4), 10 mM NADPH, 20 mM CaCl_2_, 100 μM [^3^H]-L-arginine, 1 mg/mL calmodulin, a 1:1 mixture of FAD and FMN, and 50 mM tetrahydrobiopterin (BH_4_), in a final volume of 100 μL at 37 °C for 30 min. The reaction was terminated by adding 1 mL of 0.02 M HEPES buffer (pH 5.5) containing 2 mM EDTA, 2 mM EGTA, and 1 mM L-citrulline. The reaction mixture was applied to 1 mL Dowex 50WX-8 columns (Na^+^ form) to separate [^3^H]-L-citrulline. [^3^H]-L-citrulline was measured using a Quanta Smart TriCarb Liquid Scintillation Analyzer (Packard Instrument Company, Meriden, CT, USA).

### Statistical analysis

Results are expressed as means ± S.E.M. One-way ANOVA and Duncan test were used for the statistical analysis. P<0.05 value was considered as statistically significant.

## Results

### Blood pressure and weight Parameters

**Fig. 1:**
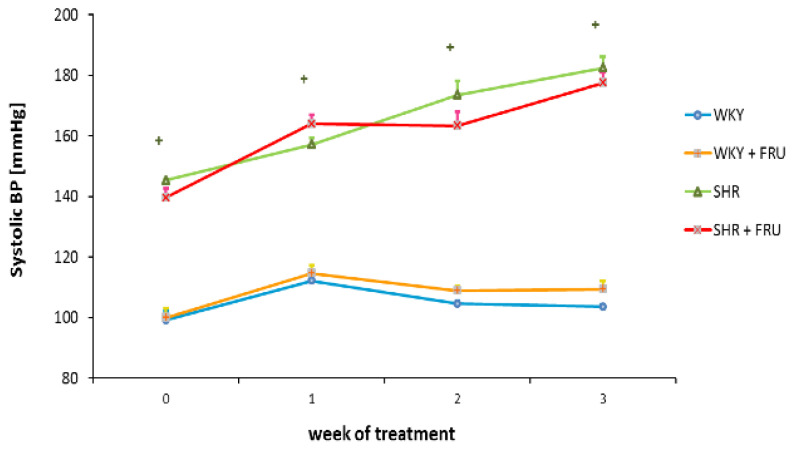
Systolic blood pressure (BP) after short-term fructose treatment in Wistar Kyoto (WKY) and spontaneously hypertensive rats (SHR). Values represent mean ± SEM. ^+^ p<0.05 WKY vs. SHR

At the end of experiment, mean systolic blood pressure after short-term treatment with 10 % fructose neither in WKY nor SHR was changed, when compared to control age-matched untreated rats (Fig. 1). Body weight did not differ between control and fructose drinking rats, however, KW/BW ratio in SHR was increased due to the fructose treatment (Table 1).

### Lipid profile

Fructose treatment decrease plasma HDL level in WKY, while increase total cholesterol, VLDL, and triglyceride levels in the plasma of SHR (Table 1).

### CD concentration

**Fig. 2:**
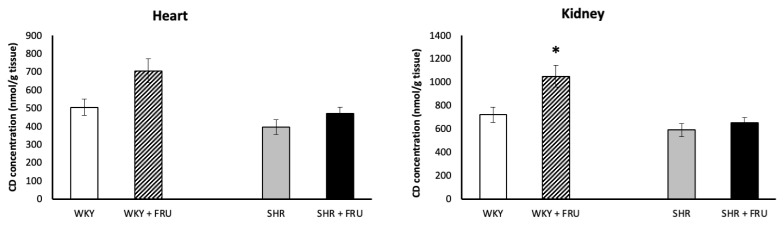
The effect of short-term treatment with 10 % fructose (FRU) on conjugated diene (CD) concentration in the heart and kidney of Wistar Kyoto (WKY) and spontaneously hypertensive rats (SHR). Values represent mean ± SEM. * p < 0.05 WKY vs. WKY+FRU

Although increasing trend in WKY, fructose treatment did not change CD concentration in the heart of both WKY and SHR. On the other hand, it increased significantly CD concentration in the kidney of WKY (Fig. 2).

### Protein expressions of NADPH oxidase, NF-κB, and SOD

**Fig. 3:**
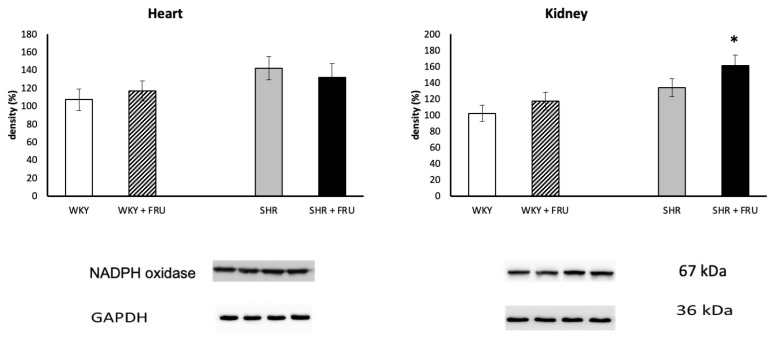
The effect of short-term treatment with 10% fructose (FRU) on protein expression of NADPH oxidase in the heart and kidney of Wistar Kyoto (WKY) and spontaneously hypertensive rats (SHR). Values represent mean ± SEM. * p < 0.05 SHR vs. SHR+FRU

**Fig. 4:**
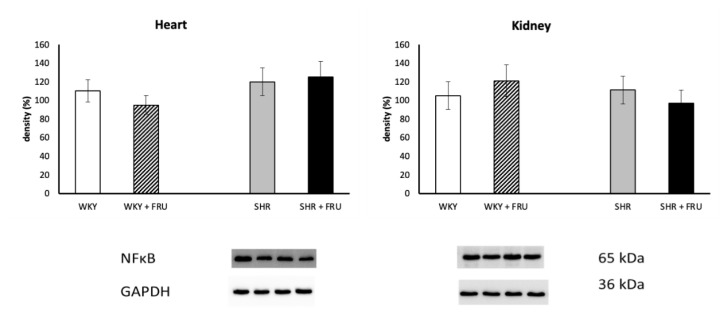
The effect of short-term treatment with 10% fructose (FRU) on protein expression of nuclear factor kappaB (NF-κB) in the heart and kidney of Wistar Kyoto (WKY) and spontaneously hypertensive rats (SHR).

**Fig. 5:**
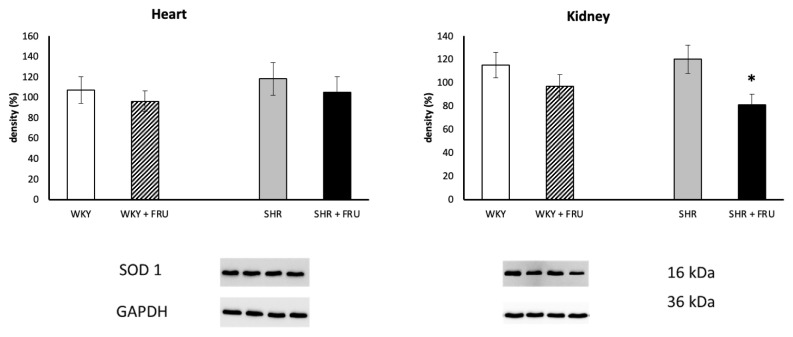
The effect of short-term treatment with 10 % fructose (FRU) on protein expression of superoxide dismutase (SOD) in the heart and kidney of Wistar Kyoto (WKY) and spontaneously hypertensive rats (SHR). Values represent mean ± SEM. * p < 0.05 SHR vs. SHR+FRU

Fructose treatment significantly increased NADPH oxidase expression in the kidney of SHR, while had no effect in the heart of both WKY and SHR (Fig. 3). Fructose treatment also did not change NF-κB protein expression in any tissue of WKY or SHR (Fig. 4). Similarly, fructose treatment did not affect SOD protein expression in the heart or kidney of WKY, however, it decreased significantly SOD protein expression in the kidney of SHR (Fig. 5).

### NOS activity

**Fig. 6:**
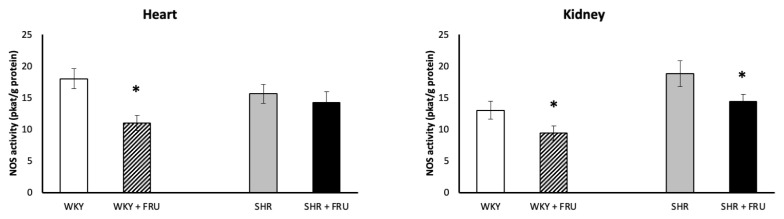
The effect of short-term treatment with 10 % fructose (FRU) on nitric oxide synthase (NOS) activity in the heart and kidney of Wistar Kyoto (WKY) and spontaneously hypertensive rats (SHR). Values represent mean ± SEM. * p < 0.05 WKY vs. WKY+FRU, resp. SHR vs. SHR+FRU

Fructose treatment significantly decreased NOS activity in the heart and kidney of WKY and in the kidney of SHR (Fig. 6).

## Discussion

In the present study, three weeks of 10 % fructose intake produced an early cardio-renal redox imbalance without affecting systolic blood pressure in either WKY or SHR rats. Specifically, fructose lowered HDL in WKY, increased total cholesterol, VLDL and triglycerides in SHR, upregulated renal NADPH oxidase and downregulated SOD in SHR, and reduced NOS activity in the heart and kidney of WKY and in the kidney of SHR. Kidney-to-body-weight ratio also increased in SHR, consistent with early renal hypertrophy. These findings indicate that even short-term, moderate fructose exposure perturbs the NO/ROS balance in a strain-dependent manner and that the hypertensive background magnifies renal susceptibility.

SHR are characterized by baseline oxidative stress and reduced NO bioavailability, which can prime the kidney for further oxidative and inflammatory hits [23–26]. Consistent with this, fructose selectively increased renal NADPH oxidase and decreased SOD in SHR (but not WKY), and reduced renal NOS activity in SHR. The absence of systolic blood pressure change is unsurprising given the relatively short exposure and moderate fructose dose. Nevertheless, the renal ROS/NOS alterations may precede hemodynamic manifestations and contribute to longer-term cardio-renal risk in the hypertensive setting. Similarly, D’Angelo *et al.* [27] showed that a high-fructose diet does not elevate blood pressure in normotensive Sprague-Dawley rats. However, Sánchez-Lozada *et al.* [28] documented increased blood pressure after 8 week-consumption of 60 % fructose in the same strain of rats and Zemancikova *et al.* [29] concluded that the increase in body adiposity due to fructose overfeeding in rats might have pro-hypertensive effect.

The kidney metabolizes and generates fructose (via the polyol pathway), with prominent KHK and GLUT5 expression in proximal tubules. This makes it exquisitely sensitive to fructose-induced ATP depletion, uric acid generation, and oxidative stress [18]. Our data on renal CD elevation in WKY and NADPH oxidase upregulation with SOD reduction in SHR fit this paradigm and suggest an early tubular redox shift toward peroxide/superoxide dominance. The observed increase in kidney-to-body-weight ratio is consistent with early hypertrophic responses to tubular stress/injury described in fructose-related renal pathology. Sánchez-Lozada *et al.* [28] reported that high fructose intake in addition to the metabolic syndrome induction can result in the development of kidney hypertrophy as well. Similarly, other experimental studies suggest fructose intake as a mechanism for kidney injury. Johnson *et al.* [30] reported that the administration of fructose (60 % diet) to rats induced renal hypertrophy with tubular cell proliferation. Similarly, Nakayama *et al.* [31] have shown that fructose, but not glucose diet, significantly increased kidney weight for 6 weeks.

It is well-known that fructose can be involved in the development of hypertriglyceridemia. Both human and animal studies have indicated that fructose consumption during several weeks can increase triglyceride levels [32,33]. Our data are in agreement with these authors. As shown in Table 1, plasma triglyceride and cholesterol levels were significantly elevated in the rats that consumed fructose compared with the control rats. From the mechanical point of view, unregulated fructolysis bypasses phosphofructokinase control and engages ChREBP/SREBP-1c programs, thereby driving VLDL-TG overproduction [34] - a pattern we observed in SHR. Ichigo *et al.* [35] even observed that high-fructose diet-induced hypertriglyceridemia is associated with increased hepatic acyl-coenzymeA:cholesterol acyltransferase 2 (ACAT2) expression.

Furthermore, our data on NADPH oxidase upregulation with concomitant SOD downregulation and reduced NOS activity argue for a deleterious net effect in SHR during early fructose exposure. Despite redox changes, NF-κB protein expression did not change. Two points may reconcile this: first, short-term fructose can elevate oxidative stress without yet producing robust transcriptional inflammation; second, our assay quantified total p65 protein, not nuclear translocation/DNA binding, which are more sensitive readouts of NF-κB activation. Longer exposure or subcellular localization assays may reveal inflammatory signaling not captured here.

The kidney exhibited greater susceptibility to fructose than the heart because proximal tubules experience higher local fructose exposure (filtered load plus polyol-pathway fructose), express abundant GLUT5/KHK-C, and rely on NADPH-dominant redox signaling [18]. Consistent with this, renal NADPH oxidase upregulation with reduced SOD shifted the renal redox tone, whereas cardiac NADPH oxidase remained unchanged and the decrease in myocardial NOS activity likely reflects NO scavenging or eNOS uncoupling rather than NADPH oxidase-driven stress [36]. The moderate dose and short duration may reveal early biochemical changes in the heart but were insufficient to trigger further remodeling [37]. However, longer high-fat-high-fructose diet in experimentally induced metabolic syndrome seems to alter the metabolism of the heart itself [38].

A decrease in NOS activity (heart and kidney in WKY; kidney in SHR) indicates compromised NO bioavailability. Multiple converging mechanisms may account for this: (i) fructose-driven uricemia directly inactivates NO, (ii) enhanced ROS (from NOX isoforms) scavenges NO, and (iii) oxidative loss of tetrahydrobiopterin leads to eNOS uncoupling, further lowering NO and increasing superoxide [9]. Although uric acid and BH4 were not measured in our study, our pattern (decrease in NOS activity with increase in lipid peroxidation/NADPH oxidase and decrease in SOD protein expression) is coherent with these mechanisms. Similarly, reduced NO bioavailability has also been reported after chronic fructose intake by other authors [39–41].

In conclusion, our study demonstrates that short-term, moderate fructose exposure induces biochemical (lipid/redox) alterations even in the absence of overt blood pressure elevation. In studied tissues, most notably the kidney, these changes are consistent with reduced NO bioavailability and a shift toward oxidative stress, mirroring the NO/ROS imbalance we documented in the heart and kidney, particularly in SHR.

## Figures and Tables

**Table 1:** The effect of short-term fructose treatment on weight parameters and lipid profile

	WKY	WKY + FRU	SHR	SHR + FRU
*BW [g]*	227.50 ± 6.56	236.50 ± 3.33	250.8 ± 4.53	241.67 ± 8.40
*HW [mg]*	814.33 ± 16.67	864.83 ± 17.04	960.67 ± 18.37	861.83 ± 10.33 **
*HW/BW [mg/g]*	3.59 ± 0.06	3.66 ± 0.08	3.68 ± 0.07	3.57 ± 0.04
*KW [mg]*	994.33 ± 18.67	1089.83 ± 18.98 xx	896.33 ± 14.06	924.17 ± 17.25
*KW/BW [mg/g]*	4.38 ± 0.08	4.61 ± 0.09	3.57 ± 0.04	3.82 ± 0.05 **
*CHOL [mmol/L]*	1.925 ± 0.08	1.785 ± 0.06	1.507 ± 0.02	1.741 ± 0.05 ^*^
*HDL [mmol/L]*	1.616 ± 0.03	1.522 ± 0.03 ^x^	1.213 ± 0.02	1.28 ± 0.04
*LDL [mmol/L]*	0.138 ± 0.01	0.093 ± 0.01	0.077 ± 0.00	0.092 ± 0.01
*VLDL [mmol/L]*	0.552 ± 0.03	0.728 ± 0.08	0.277 ± 0.01	0.740 ± 0.07 ***
*TG [mmol/L]*	1.252 ± 0.10	1.603 ± 0.17	0.686 ± 0.07	1.626 ± 0.16 ***

BW– body weight, HW – heart weight, KW – kidney weight, CHOL – cholesterol, HDL – high density lipoprotein, LDL – low density lipoprotein, VLDL – very low density lipoprotein TG – triglycerides, FRU – fructose. *Values represent mean ± SEM.*

xx p<0.01 WKY *vs.* WKY + FRU;

** p<0.01 SHR *vs*. SHR + FRU;

*** p<0.001 SHR *vs*. SHR + FRU
